# Treatment outcome and prognostic analysis of advanced large cell neuroendocrine carcinoma of the lung

**DOI:** 10.1038/s41598-022-18421-3

**Published:** 2022-10-04

**Authors:** Lu Xia, Lile Wang, Zihan Zhou, Shuhua Han

**Affiliations:** 1 Department of Respiratory and Critical Care Medicine, The Fifth People’s Hospital of Wuxi, Wuxi, 214000 China; 2 School of Medicine, Southeast University, Nanjing, 210009 China; 3 Department of Respiratory and Critical Care Medicine, Zhong da Hospital of Southeast University, Nanjing, 210009 China

**Keywords:** Cancer, Diseases, Medical research, Oncology

## Abstract

The optimal systemic treatment of advanced large cell neuroendocrine carcinoma (LCNEC) is still controversial. We intend to explore advanced LCNEC through SEER database, construct nomogram model of advanced LCNEC, and understand the effect of different treatment regimens on LCNEC. We collected 909 patients, divided them into a training set validation set, constructed nomograms using Cox proportional hazards regression models, and evaluated nomogram discrimination and calibration by C-index and calibration curves. Kaplan–Meier will also be used to compare OS in different groups of patients and to explore the impact of different treatment regimens on advanced LCNEC. On the nomogram plotted, the nomogram predicted AUC values over time were always greater than 0.7, the C-index was 0.681 (95% CI 0.656–0.706) and 0.663 (95% CI 0.628–0.698) in the training and validation sets, respectively, and patients were divided into two groups according to risk, and a significant difference in OS was observed between the high-risk and low-risk groups in the training and validation cohorts. Different treatment analyses showed that chemotherapy is still the best treatment for advanced LCNEC. This nomogram provides a convenient and reliable tool for individual assessment and clinical decision-making of patients with advanced LCNEC.

## Introduction

Large cell neuroendocrine carcinoma of the lung is a rare type of cancer in lung cancer with a high mortality rate^1^. LCNEC was first proposed in 1991 and its pathological features were described^2^. Recent studies have shown that the incidence of large cell neuroendocrine carcinoma of the lung increased substantially from 1991 to 2010^3^.

At present, it is difficult to diagnose a small sample of LCNEC, and most of them have advanced disease and metastasis at the time of diagnosis^4^. At the same time, advanced metastasis hinders the further improvement of the prognosis of patients with large cell neuroendocrine carcinoma of the lung, so the treatment and management of its advanced stage is more important^5^.

At present, there are still many conflicting results on the prognosis and treatment of large cell neuroendocrine carcinoma of the lung^6^, and surgery for early LCNEC is still the treatment of choice, but due to the lack of high-level clinical evidence, there is a lack of consensus on the optimal treatment for advanced and unresectable LCNEC. At the same time, whether radiotherapy can improve the prognosis of patients with brain metastases and other issues, due to the small number of cases, clinical diagnosis is difficult, the analysis of its prognosis still needs to be explored, so the purpose of our article is to study the relationship between the treatment of advanced LCNEC and prognosis and establish a prognostic model.

## Result

### General situation of training set and validation set population

A total of 909 patients with stage IV LCNEC from the seer database were included in this study, 331 (54.6%) were male in the training set, 410 (67.7%) underwent chemotherapy in the training set, in the validation set, the age set was 65.69 ± 11.2, 192 (63.4%) underwent chemotherapy, and the population general conditions of the training set and validation set were found in Table 1.

**Table 1:** The population general conditions of the training set and validation set.

Characteristics	Training cohort (n = 606)	Validation cohort (n = 303)
**Age**		
< 60 years	65.3 ± 10.1	65.69 ± 11.2
**Race**		
White	513 (84.7%)	251 (82.8%)
Black	71 (11.7%)	38 (12.5%)
Other	22 (3.6%)	14 (4.6%)
**Sex**		
Male	331 (54.6%)	176 (58.1%)
**Laterality**		
Left	230 (38.0%)	117 (38.6%)
Right	326 (53.8%)	161 (53.1%)
Paired	47 (7.8%)	25 (8.3%)
Only one side	3 (0.5%)	0 (0%)
**AJCC T, 7th**		
T0–T1	75 (12.4%)	38 (12.5%)
T2	149 (24.3%)	63 (20.8%)
T3	135 (22.3%)	68 (22.4%)
T4	170 (28.1%)	92 (30.4%)
Tx	77 (12.7%)	42 (13.9%)
**AJCC N, 7th**		
N0	142 (23.4%)	68 (22.4%)
N1	49 (8.1%)	28 (9.2%)
N2	260 (42.9%)	137 (45.2%)
N3	129 (21.3%)	55 (18.2%)
Nx	26 (4.3%)	15 (5.0%)
Surgery	41 (6.8%)	16 (5.3%)
Radiation treatment	322 (53.1%)	153 (50.5%)
Chemotherapy treatment	410 (67.7%)	192 (63.4%)
Bone metastasis	206 (34.0%)	83 (27.4%)
Brain metastasis	222 (36.6%)	105 (17.3%)
Liver metastasis	199 (32.8%)	96 (31.7%)

### Selection of risk factors in the training set

We screened the training set patients for independent predictive risk factors associated with prognosis from 13 risk factors using cox univariate, multivariate analysis as detailed in Table 2.

**Table 2:** Univariate multivariate analysis.

Characteristic	Variable	Univariable analysis		95% CI		Multivariable analysis		95% CI	
		P	HR	Lower	Upper	P	HR	Lower	Upper
Age	< 60 years	0.000232	0.754	0.649	0.876	0.005	0.800	0.684	0.936
Race	White	0.273				0.243			
	Black	0.459	1.139	0.807	1.607	0.377	1.172	0.824	1.665
	other	0.891	0.973	0.660	1.435	0.976	0.994	0.667	1.481
Sex	male	0.047	1.148	1.002	1.315	0.006	1.216	1.058	1.397
AJCC T, 7th	T0–T1	0.013				0.006			
	T2	0.073	0.781	0,596	1.024	0.233	0.839	0.629	1.119
	T3	0.782	0.967	0.765	1.224	0.771	1.039	0.801	1.349
	T4	0.281	1.138	0.899	1.440	0.093	1.246	0.964	1.612
	Tx	0.290	1.129	0.901	1.415	0.081	1.247	0.973	1.599
AJCC N, 7th	N0	0.002				0.001			
	N1	0.007	0.622	0.441	0.877	0.091	0.729	0.506	1.052
	N2	0.155	0.756	0.514	1.112	0.340	0.819	0.544	1.234
	N3	0.221	0.815	0.588	1.131	0.945	1.012	0.715	1.434
	Nx	0.642	0.922	0.654	1.299	0.436	1.156	0.803	1.663
Surgery		0.000077	0.554	0.413	0.742	0.020	0.696	0.513	0.944
Radiation treatment		0.012	0.841	0.735	0.963	0.046	0.852	0.727	0.997
Chemotherapy treatment		6.45E − 18	0.532	0.461	0.614	5.92E − 25	0.441	0.378	0.515
Bone metastasis		0.000003	1.413	1.222	1.633	0.000493	1.315	1.127	1.534
Brain metastasis		0.277	1.081	0.939	1.244	0.000098	1.386	1.176	1.634
Liver metastasis		1.48E − 7	1.472	1.274	1.700	5.87E − 7	1.491	1.274	1.743

### Nomogram construction and validation

**Figure 1:**
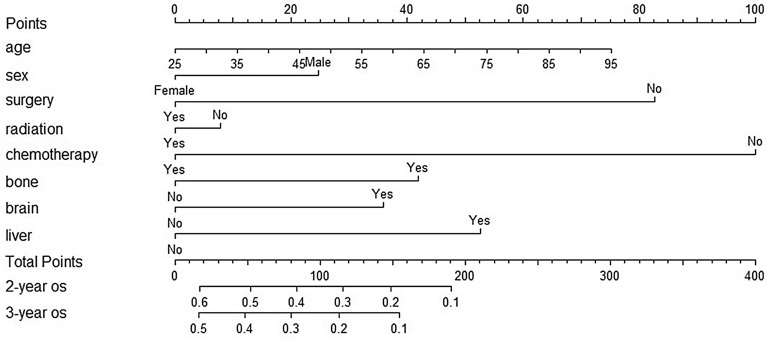
Prognostic nomogram model constructed with advanced LCNEC.

**Figure 2:**
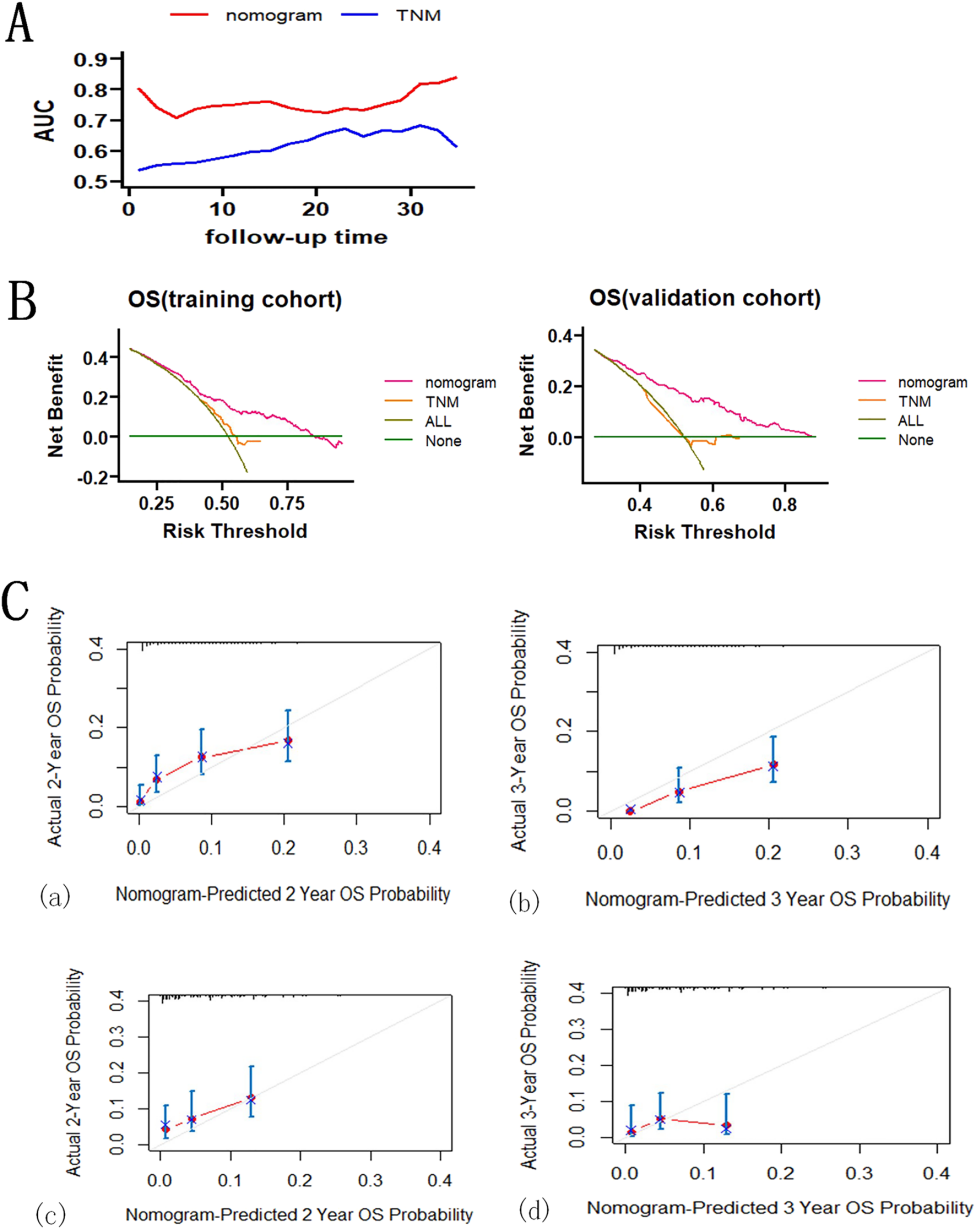
Efficacy of nomogram. (**A**) AUC curve over time for nomogram model and TNM stage. (**B**) Training set and validation set calibration model. (**C**) The DCA of the training set versus the validation set.

We constructed prognostic models for patients with advanced LCNEC, such as Fig. 1, using selected variables to demonstrate the prediction of OS at 2 and 3 years. The C-index was 0.681 (95% CI 0.656–0.706) in the training set and 0.663 (95% CI 0.628–0.698) in the validation set. We compared the nomogram and TNM analysis for the prediction of time at the same time and plotted the time AUC curve, which showed that the nomogram was significantly better than the TNM stage, and the nomogram predicted AUC value was always greater than 0.7 (Fig. 2A) over time indicating that the nomogram had good discriminatory ability.

The calibration curve of 2–3 years in the training group and the calibration curve of 2–3 years in the validation group Fig. 2B showed that the nomogram had good calibration ability. The DCA of the training set versus the validation set is shown in Fig. 2C, and the nomogram has a good clinical benefit over TNM staging.

### Risk stratification and KM survival curve based on nomogram

**Figure 3:**
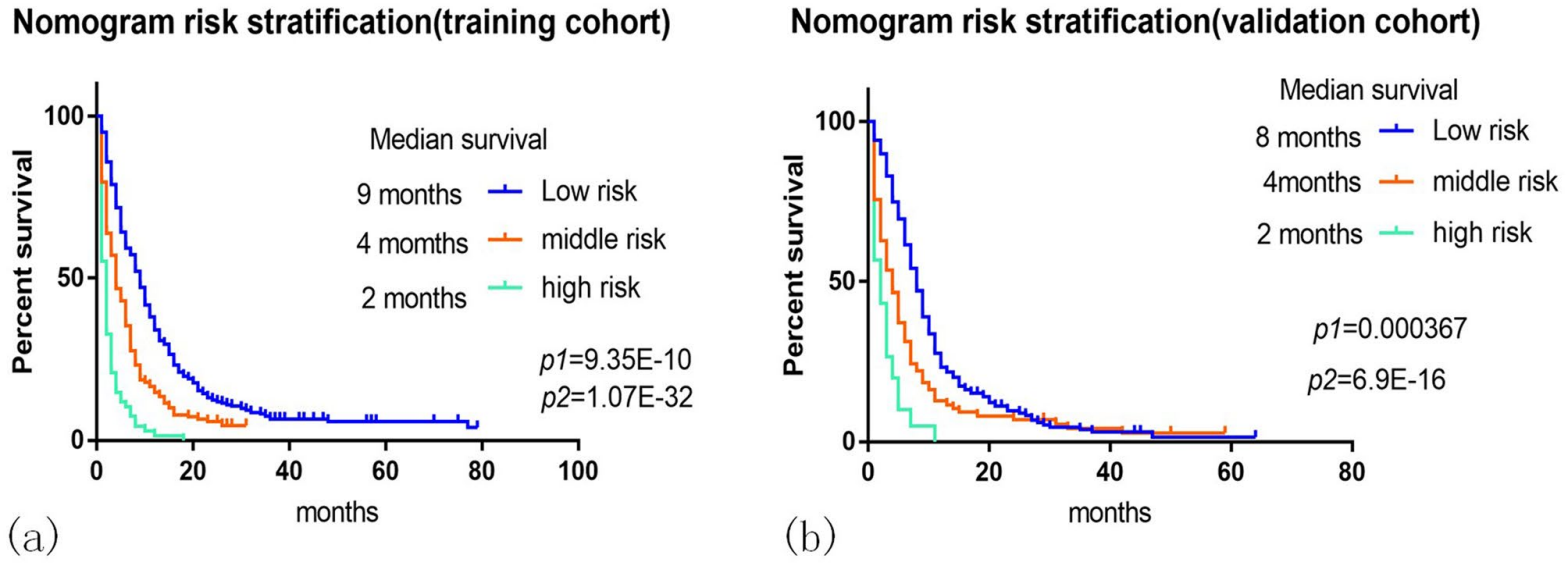
Prognostic risk score in patients in advanced LCNEC.

We classified them into low, medium, and high risk groups by x-tile software. The KM curve (Fig. 3a,b) was drawn. It could be seen in the high-risk group that the survival time of the training set and the validation set was significantly lower than that of the low-risk group. In the training set, there were statistical differences in OS between the low-risk group and the medium-risk group (P = 9.35E − 10), and there were statistical differences in OS between the low-risk group and the high-risk group (P = 1.07E − 32). Similarly, in the validation set, a significant difference in OS was also observed between the low-risk group and the medium-risk group (P = 0.000367), and between the low-risk group and the high-risk group (P = 6.9E − 16).

### Survival curves by treatment

**Figure 4:**
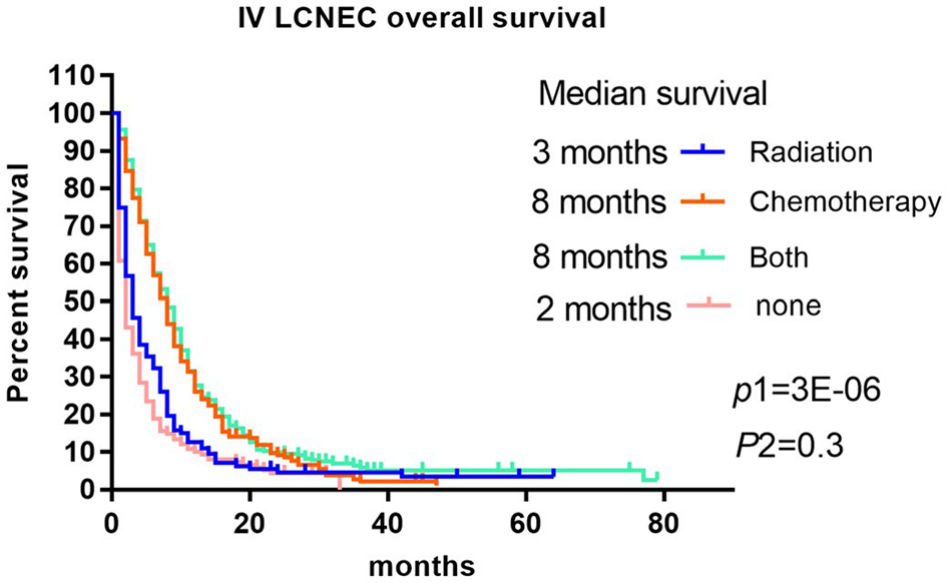
Prognosis of different treatment methods for advanced unoperated LCNEC.

Since the chance of obtaining surgery for advanced LCNEC is less, we explored the survival time of the radiotherapy only group, the chemotherapy only group, and the chemoradiotherapy group, No treatment group, such as Fig. 4.

There was a significant statistical difference between the radiotherapy only group and the chemotherapy only group (P1 = 3E − 06), and there was no significant statistical difference between the chemoradiotherapy group and the chemotherapy only group (P2 = 0.3) Thus, radiotherapy did not significantly improve the prognosis of advanced LCNEC.

## Discussion

The increased incidence of LCNEC, especially after 2008, may be related to the increasing understanding of this tumor by pathologists^6^. For patients with early LCNEC, surgical treatment is mostly used, and chemotherapy with etoposide and platinum can reduce the probability of recurrence^7^. However, for patients with advanced LCNEC, our study showed that patients in the chemotherapy only group could obtain the best survival time compared with those in the radiation only group. Although there was no significant difference in the overall survival time between the chemotherapy and chemoradiotherapy groups, excessive radiotherapy was not necessary. Large cell neuroendocrine carcinoma (LCNEC) of the lung is a highly aggressive malignant tumor, and its biological relationship with small cell and non-small cell carcinoma has been a long-standing debate^8^. There are still different controversies about whether chemotherapy are similar to small cell lung cancer or NSCLC. Current molecular biology studies on the classification of LCNEC into SCLC-like and NSLCS-like may favor the choice of chemotherapy regimen for patients with LCNEC, a study by Zhou et al. showed that etoposide–platinum therapy was superior to pemetrexed-platinum and gemcitabine/taxane-platinum doublets in response rate and survival in patients with SCLC-like LCNEC, while pemetrexed, platinum, or etoposide, platinum had longer survival than gemcitabine/taxane-platinum therapy in patients with NSCLC-like LCNEC^9^. It has also been suggested that inhibitors of δ-like Notch canonical ligands and immunotherapy may provide alternatives for tailored treatment of patients with LCNEC^10^. Regarding the treatment of LCNEC Petros et al.^11^ showed that untreated patients with stage IV LCNEC had significant T-cell repertoire alterations. Encouraging data have emerged on the efficacy of ICI in patients with advanced LCNEC, but large-scale clinical studies are still needed to find the clinical benefit and best predictive biomarkers of ICI in patients with LCNEC ^12,13^.

For the prognosis of patients with LCNEC, previous studies have shown that laterality is an independent prognostic factor in LCNEC, with fewer tumors on the left side but a worse prognosis than on the right^14^. Also in neuroendocrine tumors, angiogenic factor expression is increased, suggesting a possible prognostic marker^15^. In this paper, a prognostic prediction model for advanced LCNEC was established by nomogram, which provides a new idea for clinical work.

However, there are still some limitations in this article: (1) this study is limited by the retrospective study data collection, which may lead to inevitable bias, (2) because the SEER database does not have accurate chemotherapy drug information, the prognostic impact of different chemotherapy drugs on LCNEC is not known. (3) Seer database lacks about patient tumor markers, patient general pulmonary function and other conditions, so these factors cannot be included in the variable screening of nomogram, so more comprehensive information may still be needed to establish a more perfect nomogram. (4) Lack of external validation.

## Conclusions

We have established and validated a nomogram that provides a convenient and reliable tool for predicting survival and selecting the treatment options with advanced large cell neuroendocrine carcinoma of the lung. Chemotherapy in advanced patients is still an important treatment for its benefit. More prospective studies are still needed in the future to confirm these results.

## Materials and methods

### Data source and screening

All data are from the SEER database (Surveillance, Epidemiology, and End Results) (https://seer.cancer.gov/), which is a database with multiple cancer statistics and is the gold standard for cancer data based on population data^16^, which records 28% of the US population, reports staging and histological details of all cancers, allows the study of specific and rare cancers^17^, with the aim of reducing the cancer burden in the US population, and patient information data are downloaded in seer*stat software. SEER belong to public databases. The patients involved in the database have obtained ethical approval. All experiments conformed to the Declaration of Helsinki.

**Figure 5:**
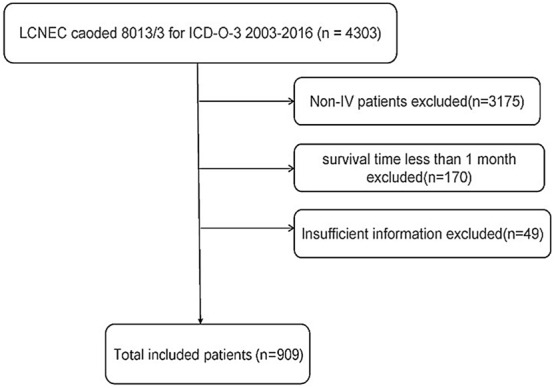
Flowchart of patient selection.

We obtained lung cancer data from 2003 to 2016 from the SEER database, while collecting Ageat diagnosis, staging, sex, Laterality, Primary Site, ICD-O-3 months, surgery condition, Radiation recode, Chemotherapy recode, Survival histology, Vital race, AJCC7theditioncondition, N stage, M stage, T stage, Bone status, and liver and brain metastasis. Inclusion criteria (1) the ICD-O-3 code of pulmonary large cell neuroendocrine carcinoma is 8013/3; (2) AJCC7theditionis stage IV, we excluded the patients with lack of data and the number of medical records with survival staging less than 1 months, the specific information check Fig. 5.

### Establishment of training set and validation set

A total of 909 patients were included and randomly divided into training set and internal validation set in a ratio of 7:3. The training set was used to screen variables and construct models, draw ROC curves and calculate model AUC values, assess model discrimination, draw calibration curves to assess model calibration, and draw decision curves to assess model clinical utility. ROC curve, AUC value, calibration curve and decision curve were calculated for the validation set to evaluate the model.

### Statistical analysis

SPSS 23.0 (IBM Inc., Chicago, IL, USA) and R software version 4.0.5 (http://www.r-project.org/) were used for statistical analyses. GraphPad prism 7 was used to plot survival curves. Univariate analysis with COX regression and multivariate analysis were used to screen variables for constructing the model. The OS curves of patients with different treatments and different risk strata were analyzed using Kaplan–Meier analysis. Nomogram, ROC curve, AUC value, C-index, calibration curve, and DCA were all implemented by R software. The values of C-index or AUC range from 0.5 to 1.0, with 0.5 representing random probability and 1.0 representing perfect ability to accurately judge results. We calculated the hazard score of patients using nomograms and divided them into low-risk, mediate-risk, and high-risk groups. Kaplan–Meier was used to compare OS between groups of patients, and the cutoff value for the total score was calculated by the X-Tile version. The results were statistically different when the P value was less than 0.05.

## Supplementary Information

Supplementary Information.

## Data Availability

All data information was obtained from the seer database, obtained by application, and downloaded in the seer stat software. All data generated or analysed during this study are included in this published article (and its Supplementary Information files).
